# Effectiveness of Narrative Messaging Styles about the Social Determinants of Health and Health Inequities in Ontario, Canada

**DOI:** 10.3390/ijerph182010881

**Published:** 2021-10-16

**Authors:** Emily Churchill, Ketan Shankardass, Andrea M.L. Perrella, Aisha Lofters, Carlos Quiñonez, Louise Brooks, Dana Wilson, Maritt Kirst

**Affiliations:** 1 Department of Psychology, Wilfrid Laurier University, Waterloo, ON N2L 3C5, Canada; chur8490@mylaurier.ca (E.C.); kshankardass@wlu.ca (K.S.); 2 Department of Health Sciences, Wilfrid Laurier University, Waterloo, ON N2L 3C5, Canada; 3 Department of Political Science, Wilfrid Laurier University, Waterloo, ON N2L 3C5, Canada; aperrella@wlu.ca; 4 Women’s College Hospital, Toronto, ON M5S 1B2, Canada; Aisha.Lofters@wchospital.ca; 5 Faculty of Dentistry, University of Toronto, Toronto, ON M5S 1A1, Canada; Carlos.Quinonez@dentistry.utoronto.ca; 6 Wellington-Dufferin-Guelph Public Health, Guelph, ON N1G 0E1, Canada; Louise.Brooks@wdgpublichealth.ca; 7 Public Health Sudbury & Districts, Sudbury, ON P3E 3A3, Canada; wilsond@phsd.ca

**Keywords:** social determinants of health, health inequities, public opinion, messaging

## Abstract

Health inequities are systemic, avoidable, and unjust differences in health between populations. These differences are often determined by social and structural factors, such as income and social status, employment and working conditions, or race/racism, which are referred to as the social determinants of health (SDOH). According to public opinion, health is considered to be largely determined by the choices and behaviours of individuals. However, evidence suggests that social and structural factors are the key determinants of health. There is likely a lack of public understanding of the role that social and structural factors play in determining health and producing health inequities. Public opinion and priorities can drive governmental action, so the aim of this work was to determine the most impactful way to increase knowledge and awareness about the social determinants of health (SDOH) and health inequities in the province of Ontario, Canada. A study to test the effectiveness of four different messaging styles about health inequities and the SDOH was conducted with a sample of 805 adult residents of Ontario. Findings show that messages highlighting the challenges faced by those experiencing the negative effects of the SDOH, while still acknowledging individual responsibility for health, were the most effective for eliciting an empathetic response from Ontarians. These findings can be used to inform public awareness campaigns focused on changing the current public narrative about the SDOH toward a more empathetic response, with the goal of increasing political will to enact policies to address health inequities in Ontario.

## 1. Introduction

Health inequities are systemic, avoidable, and unjust differences in health between social groups [1]. These differences in health are often determined by factors such as income and social status, education, race/racism, and employment and working conditions, which are referred to as the social determinants of health (SDOH) and reflect the negative impact of social, structural factors, and systems on health. While Canada spends a considerable amount of money on healthcare, in comparison to other wealthy nations, it is not developing policies or focusing energy and funding on addressing the negative impact of these social determinants to reduce health inequities [2]. In Ontario—Canada’s most populous province—there has been some action to create more equitable access to healthcare, such as strategies developed by Health Quality Ontario, but little policy development to date has focused on strengthening other SDOH [3]. For example, Ontario’s child and family poverty rates are increasing [4,5], and there remain significant unmet needs in affordable housing across Ontario, which disproportionately impacts populations who experience health inequities (e.g., those with lower incomes, recent immigrants, and single-parent households) [6].

As products of social contexts, political climate and policy greatly affect the SDOH and health inequities. It is widely recognized that policy change is the most effective way to strengthen the SDOH and reduce health inequities [2,7]. Public opinion can influence policy change, especially for issues of importance to the public [8,9]. Some recent examples of public-driven health policy change include recreational cannabis legalization in certain American states [10,11] and sustained implementation of harm reduction programming in Canada and Australia [12,13]. Shifting public opinion toward a greater understanding of the SDOH is an important mechanism in increasing the political will to act and implement policy changes for these issues. Such a task is even more urgent, given the current context of the COVID-19 pandemic, which has exacerbated health inequities among marginalized populations within Ontario and at a global level [14].

Several Canadian studies on public opinion of the SDOH and health inequities show that many Canadians overemphasize the role of the individual when attributing the causes of health outcomes [15,16,17,18,19] and that there is a lack of understanding of the role of the SDOH in shaping health status and related inequities. Attribution theory states that people attribute causes to actions or behaviours to help make sense of their surroundings [20], and, as this theory suggests, antecedents, such as information that directly influences attributions. If people do not understand the SDOH, they will not have a framework in which to attribute health outcomes to larger social causes. Herein lies the crux of the health inequity problem definition—the need to shift the perceived understanding from a framework that emphasizes individual responsibility for health to a framework that recognizes the role of social and structural factors in determining health.

Problem definitions are subjective ways of thinking about and explaining issues [21]. Altering how a problem is defined and understood by the public can result in the identification of different approaches to addressing the problem. Research has shown that the way people attribute responsibility for health outcomes translates to policies and interventions they support [15,16]. Citizens who attribute poor health to individual lifestyle factors are less likely to support solutions that focus on strengthening the SDOH [22]. Research has indicated that the best way of presenting a particular problem definition is through the deliberate use of language and rhetoric, as “use of language is critical in determining which aspect of a problem will be examined” [23] (p. 56). Problem definitions affect which issues make it to political policy agendas and what types of interventions are perceived as logical and favourable for addressing the issues [21,23]. It is, therefore, necessary to shift understandings and attributions of health outcomes and the role played by the SDOH.

The aim of this study was to determine the best ways to use language and messages as tools to change the way people understand the SDOH and health inequities, and thereby increase public support for solutions to health inequities in the province of Ontario. Previous research conducted with Ontarians showed that certain sub-populations will be more difficult to reach, in terms of increasing awareness about income-based health inequities and supporting health equity solutions [15,18], five in particular: people who identify as male, people under the age of 35, people with a conservative political affiliation, people with low socioeconomic position (i.e., low annual income, unemployment, or low educational attainment), and people who were born in Canada [15,18]. These previous findings set the groundwork for the current study, which sought to address two research questions:

1. Which message style is most effective for communicating information on the SDOH and health inequities?
2. Which message style is more effective for communicating information to sub-populations that are more difficult to reach regarding awareness and understanding of the SDOH and health inequities?

## 2. Materials and Methods

### 2.1. Message Creation

Four different narrative messages about the SDOH and health inequities were developed based on strategies used in previous health communications research conducted by Gollust et al. [24] and Niederdeppe et al. [25]. The research team developed messages by conducting a literature review and a content analysis of Canadian news media.

First, a review of the literature on strategies for developing messaging for the public about the SDOH was conducted to determine a theoretical basis on which to develop the SDOH messages. According to the literature, narrative messages are more effective than factual or statistical messages, and only one fact should be included about the SDOH in each narrative message [25,26,27], in order to have the most impact on the message readers. Narrative persuasion theory states that a narrative approach to messaging can be effective for increasing understanding of the SDOH, as well as support for related policies, by eliciting an empathetic response from the message reader [28]. Theoretically, an effective message about the SDOH should elicit empathetic responses from participants [28]. Evidence suggests that people who have not experienced the negative effects of the SDOH, such as low income or food insecurity, are less likely to support policies that mitigate and strengthen the SDOH [29]. Feelings of empathy, which involve taking the perspective of others who may not share similar experiences, can lead to feelings of discomfort. This discomfort can then motivate people to change their attitudes and to reduce inconsistency between their current and previous attitudes, as well as to take responsibility in supporting solutions. These processes, embedded within an engaging story, can produce a persuasive response, contributing to a shift in causal attributions of health [28].

A content analysis of Canadian news media was also conducted. The aim of the content analysis was to use the findings to create messages that reflected similar topics and frames, as reported in the Canadian news media, so that the messages in the study would be relevant to those reading messages. The most common SDOH topics that emerged from the content analysis were income, housing, food security, and education, and each of these topics were included in the messages developed for the study.

Previous research on public attributions of health inequities has shown that people in Ontario primarily attribute health inequities to the following: the “plight of the poor”, an empathetic, societal responsibility perspective (i.e., focusing on the impact of poverty on negative health outcomes), with 58.3% agreement among a sample from the Ontario public; the “privilege of the rich”, a societal responsibility perspective that highlights differences in privilege (i.e., focusing on the impact of wealth on positive health outcomes) (58.7% agreement); or “blame the poor”—a perspective that attributes health inequities primarily to individual choices and responsibility (43.1% agreement) [22]. The content analysis confirmed that a “blame the poor” attribution or frame is not common in Canadian news articles from the past two years, so the top two predetermined frames were included, with two of the messages written with a “plight of the poor” framing and two of the messages written with a “privilege of the rich” framing.

After several rounds of revising the messages, in partnership with a research advisory group (comprised of researchers and public health professionals), four final message types were created: (1) plight of the poor, social responsibility frame; (2) plight of the poor, hybrid social and individual responsibility frame; (3) privilege of the rich, social responsibility frame; and (4) privilege the rich, hybrid social and individual responsibility frame (Table 1). Similar to prior health communications research studies [24,25], the messages all invoked the values and emotions of the same fictional protagonist, Brian, by including quotations for readers to gain a sense of his feelings [26,30], in alignment with each of the health inequity attributions. A frame highlighting racial disparities and a solely individual responsibility frame were not included, as the literature showed that these frames can elicit negative feelings from readers [24,31]. Finally, the four messages were piloted in a focus-group style discussion to ensure that they were clear and understandable.

### 2.2. Data Collection

Data were collected from 1489 survey participants through a market-based research firm (Dynata, Shelton, CO, USA). The market-based research firm conducted a random assignment of the sample to either receive an online survey containing one of the four SDOH messages or a survey containing no SDOH messages (no message control group) (completion rate of 81%). Surveys containing the SDOH messages also contained a series of questions assessing the effectiveness of communication on health inequities, operationalized as perceived message strength, anger, and empathy (e.g., sympathy toward the main character of the message and feeling upset by the character’s situation), as well as support for different health equity policies and demographic questions. Surveys with no SDOH messages contained only a series of questions examining support for different health equity policies and demographic questions. Sample size calculations were conducted to ensure that the sample was large enough to detect statistically significant associations pertinent to the population of Ontario, as well as the identified difficult-to-reach sub-populations that were previously found to have less awareness of the SDOH and health inequities [15,18]. An estimated sample size was needed in order to include 50% male identifying participants. Assuming a response baseline risk of 0.75, based on an earlier finding that 75% of Ontarians support housing interventions [15], an odds ratio of OR = 0.65 for males in agreement with both types of broader and targeted interventions [15], as well as an alpha of 0.05, a sample size of 843 was calculated, or approximately 169 participants per message or control group. As the focus of this paper is on identifying which messages resonated most with the Ontario public, the present analysis focuses on 805 participants, comprising of the groups that read the messages (“message groups”). The no message control group participants were removed from the analysis. Differences in support for health equity policies by message and control groups were examined in a separate paper (Weatherhead et al., forthcoming). Ethics approval for the study was received from the Wilfrid Laurier Research Ethics Board (REB #5946).

### 2.3. Measures

#### 2.3.1. Dependent Variables

Two variables of message effectiveness were included in the study: *perceived message strength* and *empathy* [24,25]. *Perceived message strength* was measured based on a previously validated scale used by Gollust et al. [24]. The Likert-scale, ranging from 1 (strongly disagree) to 5 (strongly agree) includes four items: “The message is believable”, “The message is convincing”, “I agree overall with the message”, and “This message presents a strong argument”. The message strength scale was then created by averaging the four items (M = 3.66, SD = 0.84).

The dependent variable *empathy* is operationalized through measures of *sympathy* and *upset* for the message character. These variables are based on measures developed by Niederdeppe et al. [25,28] using previously validated items from the empathy response scale [32] and from Weiner’s [33] work on sympathy. The two dimensions of empathy toward the fictional message character (Brian) were *sympathy*: “How much sympathy do you have for Brian?”, with responses ranging from (1) “Hardly any” to (4) “A great deal”, and *upset by Brian’s situation*: “I felt upset for those who suffer from the problem described in this message”, with responses ranging from (1) “Strongly disagree” to (5) “Strongly agree”.

#### 2.3.2. Independent Variables

A *survey message* variable was created to examine the effect of reading the different messages on the dependent variables. This variable was coded 1 for message 1, 2 for message 2, 3 for message 3, and 4 for message 4. In order to determine the effectiveness of the messages with difficult-to-reach subpopulations, we examined the role of gender identity, age, political affiliation, and socio-economic position [18]. A dummy variable for male gender identity was created, with male gender identity coded as 1 and all other gender identity responses (female, transgender male, transgender female, non-binary, gender variant/non-conforming, not listed, and prefer not to say) coded as 0 and used as the reference group. While previous research indicates that people over the age of 55 have more knowledge about the SDOH than younger age groups [18], our sample was younger in age, so the age groups of 35–44, 45–54, 55–64, and 65+ were coded as 1, and all age groups under 35 years old (including 18–24 and 25–34) were coded as 0 and used as the reference group. A conservative voter dummy variable was created, with conservative voters coded as 1 and all other political affiliations (NDP, liberal, and other) coded as 0 (as the reference group). Country of origin was also examined, with born in Canada coded as 1 and born in a country other than Canada coded as 0 and used as the reference group.

The role of socio-economic position (SEP) was also examined. A low SEP dummy variable was created (to avoid multi-collinearity between socio-economic related variables), by first creating an SEP composite variable consisting of annual household income, employment status, and educational attainment. Participants were considered to have a low SEP if two or more of the following conditions were met: annual household income of <$40,000; an employment status of part-time or unemployed (as opposed to full time, retired, students, or other); and an educational attainment of some high school or graduated high school (as opposed to some college or university, graduated college or university, some graduate school, or graduated graduate school).

### 2.4. Data Analysis

To address Question 1, Pearson’s chi-square tests and ANOVAs were used to examine reactions (e.g., perceived message strength and empathy) by the different survey message types. To further explore Questions 1 and 2, logistic regression analyses, using interaction terms to test for effect modification, were conducted to examine predictors of reactions to the four message types. Prior to running the multivariate logistic regression model, collinearity tests were run to check the logistic regression assumption of no multicollinearity between the predictor variables and no multicollinearity was found. To mitigate the fact that there were eight predictor variables and interaction terms in the regression models, a backwards stepwise regression model was used to derive the final parsimonious models, while avoiding overfitting the data [33]. Several multivariate logistic regression models were run to find a best fit for the data. With each of the dependent outcome variables (sympathy and upset), the analysis began with the full model, including all of the theorized predictor variables (male, younger than 25, conservative, Canadian born, and low SEP), then scaled the model back. To address Question 2, interaction terms were entered to see if the significant predictors from the main effects multivariate models were still significant when interacting with each message type. Gender identity and message type interaction terms, as well as age and message type interaction terms were entered into these models. Non-significant variables were then removed one at a time to create a final parsimonious model that is the best fit for the data [34].

## 3. Results

Demographics of the study sample are reported in Table 2. Forty percent of participants were aged 55+, 49% identified as male, and the majority lived in an urban area (84%). Seventy-five percent were born in Canada, and the majority had completed college or university (59%) and were employed (90%). The sample of 805 participants used for these analyses was representative of the Ontario population, according to 2016 Census data, in terms of gender identity, residence (urban vs. rural), and annual household income [35]. There are some differences, though, with the study sample being younger, having higher unemployment rates, a higher percentage of liberal voters, a higher percentage of participants born outside of Canada, and a higher percentage of people either in post-secondary school or completed post-secondary school compared to the Ontario population.

To examine the effectiveness of the messages, an ANOVA of message strength and chi-square tests of empathy (operationalized as sympathy for Brian and upset by Brian’s situation) were conducted by message type. The ANOVA for message strength by message type (*p* = 0.152, full results not shown) was not significant, suggesting that message type is likely not a strong predictor of perceived message strength in this study. Chi-square tests were run with the message type variable by sympathy and with message type by upset, both of which were significant. The chi-square results for sympathy and upset by message type suggested that more participants who read Message 2 (“plight of the poor”, hybrid social/individual responsibility) agreed that they felt empathy for Brian than the other three message types. Among those who read Message 2, 70% responded with both sympathy towards Brian (X^2^ = 8.35, *p* = 0.039) and were upset by Brian’s situation (X^2^ = 9.73, *p* = 0.021), compared to 54–67% of those who read the other messages (Table 3).

To examine which message style is more effective for communicating information to sub-populations that are more difficult to reach logistic regression models, indicating main effects (Models 1 and 3) and interaction effects (Model 2a,b) of predictors of sympathy and upset by Brian’s situation were run. Model 1 shows that Message 2 (“plight of the poor”, hybrid) is a significant predictor of upset, with people who read Message 2 being 1.5 times more likely to respond as upset for Brian’s situation than people who read Message 1 (reference group: plight of the poor, social) (OR = 1.5, *p* = 0.043, 95% CI [1.01, 2.38]) (see Table 4). Additionally, participants identifying as male were less likely than all other gender identities to feel upset by the character’s situation (OR = 0.55, *p* < 0.001, 95% CI [0.41, 0.75]) and younger people were almost two times more likely than older age groups to respond with upset (OR = 1.88, *p* < 0.001, 95% CI [1.38, 2.55]). Model 2a includes gender identity and message type interaction terms, while Model 2b includes age and message type interaction terms. None of the interaction terms significantly added to the models, so Model 3 was created through a backwards stepwise process, in which non-significant subgroup variables were removed one at a time. In Model 3, Message 2 remained significant (OR = 1.51, *p* = 0.054, 95% CI [0.99, 2.30]), as well as the age group (OR = 1.87, *p* < 0.001, 95% CI [1.38, 2.52]) and male gender identity (OR = 0.53, *p* < 0.001, 95% CI [0.40, 0.72]).

Table 5 presents logistic regression models indicating the main effects (Models 1 and 2) and interaction effects (Model 3a,b) of predictors of sympathy for Brian. Of the three message types, Message 2 (“plight of the poor”, hybrid) is a significant predictor of sympathy, with people who read Message 2 almost twice as likely to respond with sympathy for Brian than people who read Message 1 (plight of the poor, social) (OR = 1.69, *p* = 0.016, 95% CI [1.10, 2.60]). Again, male gender identity and age group were the only other significant predictors of sympathy in Model 1. Participants identifying as male were less likely than all other gender identities to feel sympathy for Brian (OR = 0.75, *p* = 0.065, 95% CI [0.55, 1.02], while younger people were more likely than the older age groups to respond with sympathy (OR = 2.26, *p* < 0.001, 95% CI [1.66, 3.08]). No other subpopulation variables significantly added to the model. Non-significant subgroup variables were removed one at a time to create Model 2, and, in that model, Message 2 (“plight of the poor”, hybrid) remains significant (OR = 1.69, *p* = 0.016, 95% CI [1.10, 2.57]), age group remains significant (OR = 2.37, *p* < 0.001, 95% CI [1.75, 3.20]), and male gender identity became a slightly more significant predictor of sympathy (OR = 0.72, *p* = 0.031, 95% CI [0.53, 0.97]).

Models with interaction terms were then created to examine whether the effects of each message type were moderated by age group (3a) and gender identity (3b). In Model 3a, Message 2 (“plight of the poor”, hybrid) is a significant predictor, along with the main effects of age group and gender identity. However, none of the message * age group interaction terms were significant predictors. In Model 3b, Message 2 (OR = 2.02, *p* = 0.018, 95% CIs [1.13, 3.64]) and Message 3 (“privilege of the rich”, social) are significant predictors (OR = 2.09, *p* = 0.015, 95% CIs [1.15, 2.78]). There are two significant interaction effects, both in Model 3b: Message 3 * Male and Message 4 * Male . To interpret the direction and strength of the prediction, we calculated the EXP(β) values of Message 3 * Male and found that male-identified participants who read Message 3 were less likely to respond with sympathy (OR = 0.88, *p* = 0.028). Similarly, when the EXP(β) values of Message 4 * Male were calculated, male-identified participants who read Message 4 (“privilege of the rich”, hybrid) were less likely to respond with sympathy (OR = 0.66, *p* = 0.043).

## 4. Discussion

This study sought to determine the best ways to use language and messages as tools to increase participants’ understanding of the SDOH and health inequities by eliciting a sense of empathy for those whose health is negatively impacted by social and structural factors. Results show that Message 2, which focused on the “plight of the poor”, combined with an individual/social responsibility hybrid frame, garnered the most empathetic responses, significantly more so than the other three message types, across the entire sample. Message 2 had a significant relationship with both types of empathetic responses—sympathy toward Brian and upset by Brian’s situation. Message 2 was also a significant predictor of both sympathy and upset across all logistic regression models.

These findings align with findings in previous studies. “Plight of the poor” was one of the most frequently occurring health inequity frames that emerged from the content analysis of Canadian news media. This suggests that Canadian media frequently frames health inequity in this way; therefore, these frames of understanding health inequity may resonate more with Ontarians. It is also possible that respondents would feel more empathy toward the character and the character’s situation when the message is framed to highlight social disadvantages that the poor experience, as opposed to framed as due to advantages that the rich experience. Additionally, other studies have found that including an element of individual responsibility, such as the hybrid frame in Message 2, is most effective in eliciting empathy [24,28]. Introducing an individual responsibility component to the messages may have made the messages more believable, but also made it easier for participants to empathize with the character because his values (reflective of individuality) align with their own. This framework reflects research on health communications, which suggests that appeals to positive emotions, such as empathy, dampen the reactions of anger and resistance to messages [26,30,36].

To understand which message style is more effective for communicating information to subpopulations that are more difficult to reach regarding awareness and understanding of health inequities, logistic regression models were fit to the data and observed the interaction effects between significant subpopulation predictor variables (age group and gender identity) and each of the message types (Messages 2–4). Despite finding the main effects of both gender identity and age group in predicting responses of both sympathy and upset, there were no significant interaction effects within the upset models or within the sympathy and age group model.

While two significant interaction effects were found in the model examining interactions between sympathy and gender identity (Model 3b), the results did not show any of the message types as more effective for people who identify as male but highlighted a negative effect of the “privilege of the rich” messages on sympathetic responses among male-identified participants. This further supports our findings that a “plight of the poor” frame may be more effective for evoking empathetic responses for subpopulations considered difficult-to-reach. It also suggests that messages that highlight the privilege of the rich may not resonate with a male-identified audience. These findings could be used to inform the development of public awareness campaigns (e.g., Message 2 informing the development of radio and television ads; social media videos) to help shift the public narrative regarding the SDOH and heath inequities toward a more empathetic response, with the goal of promoting greater advocacy and policy action.

### Limitations

There are several commonly cited limitations of using an online quantitative survey as a method of data collection [37]. Despite conscious testing for reliability and validity, there may be items that do not work well. If a question is misinterpreted by a large number of respondents, this will decrease the reliability of the item and the validity of the survey. This is especially a problem when using a survey, because there is no option to probe respondents. Another limitation relates to the recruitment process. While there are many benefits to using an outside research firm to recruit participants, this could also create an unrepresentative sample, as all participants will inherently have something in common—their affiliation with the firm. For example, the age distribution in the sample was not representative of the province of Ontario. Additionally, in the context of this specific project, the representativeness of the sample may be limited, as the survey was only in English, thus omitting non-English participants. Social desirability bias may affect participants’ responses, particularly for items to which responses may insinuate laying blame on individuals for health inequities. The message content reflected the experiences of SDOH of only one identity—that of a white, male character. Given the privilege associated with this identity, this character’s narrative may not have resonated with all participants. Future research should examine the effectiveness of SDOH narratives of diverse identities on eliciting empathy and support for solutions to health inequities.

The goal of this work is to contribute to the development of messages that will increase Ontarians’ awareness and empathy about the SDOH and health inequities in the province to ultimately shift public opinion and increase political will surrounding health equity policy changes. Ideally, a narrative change will lead to attitudinal change, which will lead to health policy change. It is recognized that this is not a short-term process and that raising critical consciousness about the SDOH and changing attitudes and attributions of health inequity may not lead directly to attitude change and policy change without a good knowledge translation and exchange plan to encourage political action. Furthermore, a public awareness campaign alone may not lead to narrative change and additional interventions may be necessary, accompanied by rigorous evaluation.

However, even if a shift in public opinion and problem definition of health inequity occurs, there may still be barriers to policy change. Greater public awareness does not necessarily mean that governments will act. The concept of the SDOH is not novel, yet there is little direct policy in place to reduce health inequities in Ontario [10,38]. This lack of action could be the result of many barriers. For example, there may not be enough existing evidence about what changes to policy work to decrease health inequity or there are other actors within the health sector that have more power over policy decisions than the general public [38]. Some literature suggests that “reframing social inequality as a problem of health medicalizes the problem of inequality, making it seem less amenable to systemic or structural solutions” [38] (p. 656). It is a difficult balance to strike; framing health inequity as solely the responsibility of individuals will not result in policy that eliminates negative effects of social determinants on health, while framing health inequity as a social problem can make the issue seem difficult to solve. Future research should explore the effectiveness of these messages for raising awareness of SDOH and solutions to health inequities among policy-makers.

However, it has been documented that political will is often needed to make policy changes, and there is literature to support the need for a public understanding of a problem before political engagement and policy change can take place [2,7,39,40,41,42], as well as literature to show the connection between narrative change, attitudinal shifts, and policy change [43,44]. Considering past work on public opinion in Ontario around SDOH and health inequity, it is clear that there needs to be a general shift away from the individualistic ideals of citizens to a collective view of health through increased awareness of the effects of SDOH before there will be any public traction behind SDOH related health policy changes [3,7,15]. It is noted that the connection between awareness and policy change is indirect and should be framed as so.

## 5. Conclusions

The WHO [7] has stated that addressing the SDOH and health inequities is an ethical obligation. By definition, health inequities are avoidable and, therefore, unjust. Despite the evidence of the effects of the SDOH on health outcomes, Ontario is not allocating enough resources toward strengthening the SDOH and reducing health inequities [2]. Changes to policy are considered to be the best way to decrease the negative effects of the SDOH [2,7]. Among a sample of Ontarians, over half believed that everyone in the province had an equal chance at a healthy life, but that the government had no role to play in addressing health inequity [15], suggesting a lack of awareness of the SDOH, resultant health inequities, and the critical role that government policy-making can play in addressing these issues [2,7]. Raising awareness through narrative change techniques has been shown to contribute to shifts in attitude and subsequent policy change [43,44]. Our findings suggest that the use of a hybrid message emphasizing both individual and social responsibility for health may be effective for eliciting empathetic responses from the public. Public awareness campaigns on the SDOH and health inequities that highlight the experiences of marginalized populations through narrative messaging, similar to that tested in this study, could be an important step in increasing the political will, which is necessary to implement policies and programming that will address these issues. This action is even more imperative in the current context of the COVID-19 pandemic, when marginalized populations are most affected by the virus and its impacts [14,45]. Attempting to shift the dominant narrative of individualistically determined health has the potential to decrease victim-blaming and increase the public’s recognition of the larger social structures influencing health, while putting pressure on governments to act and address health inequities.

## Figures and Tables

**Table 1:** SDOH Message Themes.

Message	Attribution of Health Inequities	Attributions of Health Outcomes	Message Thematic Summary
Message 1	“Plight of the Poor”	Social responsibility	Brian is described as a 35-year-old white male who lives and works in Ontario, Canada. He has a high school diploma but did not attend college or university, due to low-income and family circumstances. His hours at work were recently reduced, due to company downsizing, and he experiences food insecurity, lower access to resources, and poor health.
Message 2	“Plight of the Poor”	Hybrid social/individual responsibility	Brian is a 35-year-old white male who lives and works in Ontario, Canada. He has a high school diploma but never attended college or university, due to low-income and family circumstances. His hours at work were recently reduced, due to company downsizing, and he experiences food insecurity, lower access to resources, and poor health. Brian primarily eats fast food, and he begins smoking again to cope with his stressful life circumstances, exacerbating his health problems.
Message 3	“Privilege of the Rich	Social responsibility	Brian is described as a 35-year-old white male who lives and works in Ontario, Canada. He has a high school diploma but never attended college or university, due to low-income and family circumstances. His hours at work were recently reduced, due to company downsizing, and he experiences food insecurity and poor health. His experience is contrasted with his wealthy friend Pat, who has a university education, well-paying job, and experiences better health.
Message 4	“Privilege of the Rich”	Hybrid social/individual responsibility	Similar to Message 3, Brian’s circumstances are explained, but his experiences with the social determinants of health are contrasted with his wealthy friend Pat, who has a university education, well-paying job, and experiences better health. Details of Pat’s health choices are provided, as well as a statement that every time they are together, Pat tells Brian that he needs to start taking better care of himself by making healthier choices.

**Table 2:** Descriptive characteristics of sample (*n* = 805).

		Sample % (*n*) *
**Age Group**	18–34	34.5 (277)
	35–54	25 (201)
	55+	40.5 (325)
**Gender Identity**	Male	49.1 (394)
	Female	48.6 (390)
	Other gender identities	2.3 (19)
**Residence**	Urban	84.3 (675)
	Rural	15.7 (126)
**Place of birth**	Canada	75.8 (606)
	Outside of Canada	24.2 (193)
**Annual household income < $40,000**	Yes	33.4 (269)
	No	66.6 (536)
**Educational attainment**	Some high school	5.5 (44)
	Graduated high school	16.6 (134)
	Some college or university	19.1 (153)
	Completed college or university or further education	58.8 (471)
**Currently unemployed**	Yes	10.4 (84)
	No	89.6 (721)
**If the election were being held today**	Liberal	34 (271)
**would vote:**	New democratic party	22.4 (178)
	Progressive conservative	24 (191)
	Other	19.6 (156)
**Self-rated health**	Poor	3.6 (29)
	Fair	20 (160)
	Good	42 (337)
	Very good	24.2 (194)
	Excellent	10.2 (82)
**Knowledge and understanding of the**	Poor	6.1 (49)
**health issues affecting Ontarians**	Fair	28.1 (225)
	Good	43.4 (348)
	Very good	17 (136)
	Excellent	5.5 (44)

* *n*’s vary due to missing data.

**Table 3:** Indicators of upset and sympathy for health inequities messages.

Message Type	Upset by Situation %	Sympathy for Brian %
Plight, social	61%	60%
Plight, hybrid	70%	71%
Privilege, social	61%	67%
Privilege, hybrid	54%	59%
Chi-square	X^2^ (3) = 9.73	X^2^ (3) = 8.35
*n*	796	797
*p*	0.021	0.039

**Table 4:** Logistic Regression Models, predictors of upset by Brian’s situation.

		Upset by Brian’s Situation (Odds Ratios and 95% CIs)			
	Reference	Model 1	Model 2a	Model 2b	Model 3
**Message Type**					
2: Plight, hybrid	Message 1	1.56* (1.01, 2.38)	1.21 (0.66, 2.12)	1.72 ^†^ (0.91, 3.28)	1.51 ^†^ (0.99, 2.30)
3: Privilege, social	Message 1	0.99 (0.66, 1.50)	1.04 (0.57, 1.88)	0.98 (0.52, 1.86)	1.02 (0.68, 1.54)
4: Privilege, hybrid	Message 1	0.79 (0.52, 1.18)	0.70 (0.39, 1.24)	0.96 (0.51, 1.78)	0.79 (0.53, 1.18)
**Age**					
Under 35 years	35 years and older	1.88 *** (1.38, 2.55)	1.87 *** (1.39, 2.52)	2.12 * (1.18, 3.82)	1.87 *** (1.38, 2.52)
Message 2 * Under 35				0.79 (0.34, 1.86)	
Message 3 * Under 35				1.07 (0.46, 2.47)	
Message 4 * Under 35				0.71 (0.31, 1.62)	
**Gender identity**					
Male	Other gender identity	0.55 *** (0.41, 0.75)	0.46 ** (0.25, 0.81)	0.53 *** (0.39, 0.71)	0.53 *** (0.40, 0.72)
Message 2 * Male			1.53 (0.66, 3.55)		
Message 3 * Male			0.98 (0.43, 2.24)		
Message 4 * Male			1.28 (0.57, 2.90)		
**Political Affiliation**					
Conservative	NDP, liberal, other	0.84 (0.59, 1.19)			
**Nationality**					
Canadian	Not born in Canada	1.00 (0.70, 1.41)			
**SEP**		1.10 (0.78, 1.55)			
Low	High				

*** *p* < 0.001, ** *p* < 0.01, * *p* < 0.05, ^†^ *p* < 0.1.

**Table 5:** Logistic Regression Models, predictors of sympathy for Brian.

		Sympathy for Brian (Odds Ratios and 95% CIs)			
	Reference	Model 1	Model 2	Model 3a	Model 3b
**Message Type**					
2: Plight, hybrid	Message 1	1.69 * (1.10, 2.60)	1.69 * (1.10, 2.57)	1.85 * (1.05, 3.27)	2.02 * (1.13, 2.64)
3: Privilege, social	Message 1	1.34 (0.88, 2.04)	1.37 (0.90, 2.08)	1.70 ^†^ (0.97, 2.98)	2.09 * (1.15, 3.78)
4: Privilege, hybrid	Message 1	0.98 (0.65, 1.48)	1.00 (0.66, 1.51)	1.15 (0.67, 1.99)	1.47 (0.83, 2.62)
**Age**					
Under 35 years	35 years or older	2.26 *** (1.66, 3.08)	2.37 *** (1.75, 3.20)	1.82 * (1.02, 3.26)	2.32 *** (1.72, 3.14)
Message 2 * Under 35				1.26 (0.54, 2.94)	
Message 3 * Under 35				1.65 (0.71, 3.84)	
Message 4 * Under 35				1.39 (0.61, 3.18)	
**Gender identity**					
Male	Other gender identity	0.75 ^†^ (0.55, 1.02)	0.72 * (0.53, 0.97)	0.73 * (0.54, 0.98)	1.20 (0.67, 2.15)
Message 2 * Male					0.67 (0.29, 1.55)
Message 3 * Male					0.42 * (0.18, 0.97)
Message 4 * Male					0.45 ^†^ (0.20, 1.02)
**Political Affiliation**					
Conservative	NDP, liberal, other	0.88 (0.62, 1.26)			
**Nationality**					
Canadian	Not born in Canada	1.02 (0.72, 1.45)			
**SEP**					
Low	High	1.19 (0.84, 1.68)			

*** *p* < 0.001, * *p* < 0.05, ^†^ *p* < 0.1.

## Data Availability

The datasets analyzed during the current study are not currently publicly available but are available from the corresponding author on reasonable request.
